# New insights into the pathophysiology of post-stroke spasticity

**DOI:** 10.3389/fnhum.2015.00192

**Published:** 2015-04-10

**Authors:** Sheng Li, Gerard E. Francisco

**Affiliations:** ^1^Department of Physical Medicine and Rehabilitation, University of Texas Health Science Center at HoustonHouston, TX, USA; ^2^NeuroRehabilitation Research Laboratory, NeuroRecovery Research Center, TIRR Memorial Hermann Research CenterHouston, TX, USA

**Keywords:** spasticity, stroke, pathophysiology, brainstem, reticulospinal pathways

## Abstract

Spasticity is one of many consequences after stroke. It is characterized by a velocity-dependent increase in resistance during passive stretch, resulting from hyperexcitability of the stretch reflex. The underlying mechanism of the hyperexcitable stretch reflex, however, remains poorly understood. Accumulated experimental evidence has supported supraspinal origins of spasticity, likely from an imbalance between descending inhibitory and facilitatory regulation of spinal stretch reflexes secondary to cortical disinhibition after stroke. The excitability of reticulospinal (RST) and vestibulospinal tracts (VSTs) has been assessed in stroke survivors with spasticity using non-invasive indirect measures. There are strong experimental findings that support the RST hyperexcitability as a prominent underlying mechanism of post-stroke spasticity. This mechanism can at least partly account for clinical features associated with spasticity and provide insightful guidance for clinical assessment and management of spasticity. However, the possible role of VST hyperexcitability cannot be ruled out from indirect measures. *In vivo* measure of individual brainstem nuclei in stroke survivors with spasticity using advanced fMRI techniques in the future is probably able to provide direct evidence of pathogenesis of post-stroke spasticity.

## Introduction – Spasticity Represents a Phenomenon of Abnormal Plasticity

Spasticity is a common complication of stroke, but is only one of the many consequences of the UMN syndrome. It is considered a “positive” UMN sign since it represents excessive muscle tone and stretch reflex. Other so-called positive consequences include clonus and spasms. “Negative” consequences of the UMN syndrome, on the other hand, include weakness, impaired coordination, impaired motor control/planning, and easy fatigability (Mayer and Esquenazi, 2003). Following an UMN lesion, these positive and negative consequences emerge, evolve, and interact with each other, resulting in a dynamic clinical presentation during the recovery phase after a stroke (Gracies, 2005a,b). For example, weakness and spasticity often result in immobilization of a joint at a shortened muscle length, and thus potentiating contracture. This in turn exacerbates spasticity in these muscles. Such a vicious cycle continues and worsens the condition if not effectively interrupted (O’Dwyer et al., 1996; Gracies, 2005a,b). There is a wide range of the prevalence of spasticity (Wissel et al., 2013), from 19% (Sommerfeld et al., 2004) to 92% (Malhotra et al., 2011) and variable onset time after stroke (Ward, 2012).

**FIGURE 1:**
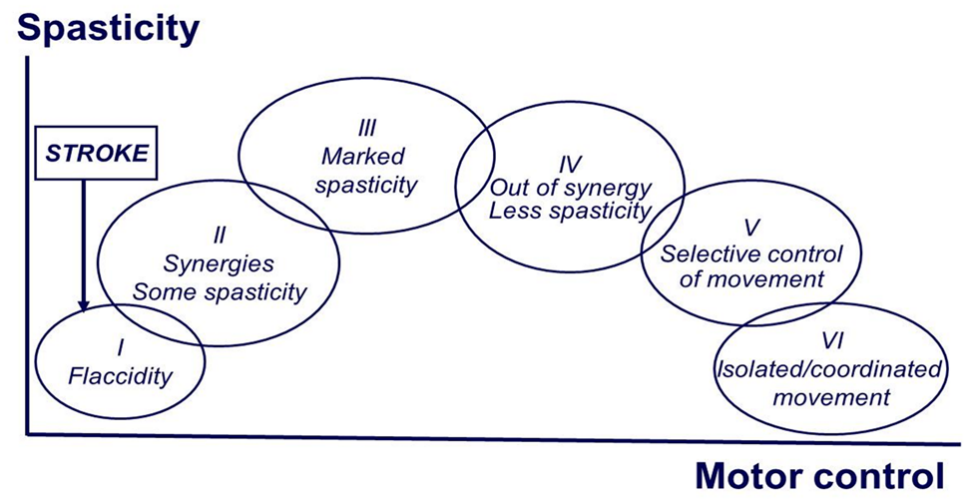
**Brunnstrom stages of motor recovery after stroke (at the end of the manuscript)**.

Motor recovery starts immediately after stroke onset, and follows a relatively predictable pattern, regardless of stoke type (hemorrhagic or ischemic, cortical or subcortical; Twitchell, 1951). Brunnstrom (1966, 1970) empirically described the stereotypical stages of motor recovery, starting with flaccidity to full recovery of motor function (see **Figure 1**). During the course of motor recovery, stroke survivors could progress from one recovery stage to the next at variable rates, but always in an orderly fashion and without omitting any stage. However, recovery may be arrested at any one of these stages (Twitchell, 1951; Brunnstrom, 1970).

Based on Brunnstrom’s description, the evolution of motor recovery appears to parallel the emergence and eventual disappearance of spasticity. The common observation is that right after stroke onset, a state characterized by depression of strength, control, and reflexes sets in. This is followed by a gradual return of reflexes, and in some cases, development of hyperreflexia. There is no a sudden change to hyperreflexia. The emergence of spasticity, though highly variable, is usually shortly following the onset of stroke (Ward, 2012). This implies that there must be some sort of neuronal plastic changes after the initial injury. This process occurs at any time, but is usually seen between 1 and 6 weeks after the initial injury (Balakrishnan and Ward, 2013). This process of plastic rearrangement often results in muscle overactivity and hyperreflexia, thus spasticity (Farmer et al., 1991). In a recent longitudinal study that examined the time course of development of spasticity and contractures at the wrist 6 weeks after a stroke, the authors reported that patients who recovered arm function showed signs of spasticity at all assessment points but did not develop contractures. In contrast, patients who did not recover useful arm function had signs of spasticity and evidence of contracture formation over the course of a 36-weeks follow-up (Malhotra et al., 2011). Collectively, emergence and disappearance of spasticity during the course of complete motor recovery imply that the development of spasticity reflects a phenomenon of abnormal plasticity. Conversely, spasticity may persist if plastic rearrangement and recovery are arrested.

## Definition

Spasticity is easily recognized, but to accurately define it is not easy. Lance (1980) proposed a consensus definition at a conference as follows:

*“Spasticity is a disorder of the sensorimotor system characterized by a velocity-dependent increase in tonic stretch reflexes* (*‘muscle tone’*)* with exaggerated tendon jerks, resulting from hyperexcitability of the stretch reflex, as one component of the upper motoneuron syndrome”*

(*Lance, 1980*).

This definition, though widely used, has been challenged, and others have proposed different descriptions (Young, 1994; Pandyan et al., 2005). However, all these definitions have explicitly emphasized that spasticity and associated phenomena are caused by abnormal or hyperexcitable spinal reflexes.

## Abnormal Regulation of Spinal Stretch Reflex and Spasticity

Excitability of spinal stretch reflex arc is maintained by a balanced descending regulation from the inhibitory dorsal RST and facilitatory medial RST and VST, as well as intraspinal processing. Therefore, hyperexcitability of stretch reflex in stroke survivors with spasticity could be mediated by two categories of mechanisms: abnormal descending regulations and/or abnormal intraspinal processing of stretch reflex.

### Abnormal Intraspinal Processing

Abnormal intraspinal processing in patients after stroke has been well documented in the literature (Gracies, 2005b; Nielsen et al., 2007; Mukherjee and Chakravarty, 2010; Burke et al., 2013). In summary, abnormal intraspinal processing could result from: (1) increased afferent input to spinal motoneurons. The sensitivity of spindles (group Ia primary and group II secondary afferent fibers) is enhanced through activation of the gamma fusimotor system and/or adaptive changes after immobilization, resulting in increased gain of stretch reflex; (2) altered inter-neuronal reflex circuits resulting in enhanced motoneuronal excitability, including reduction in presynaptic inhibition on Ia afferents, group Ib facilitation (instead of inhibition), group II facilitation, reduced reciprocal inhibition. These changes result in less inhibition from intraspinal reflex circuits on spinal motor neurons, such that motoneurons are at subthreshold or at spontaneous firing; and (3) changes in intrinsic properties of the spinal motor neurons. Disruption of descending inputs could cause spinal motoneurons to activate voltage-dependent persistent inward currents (Heckmann et al., 2005), which can lead to the development of plateau potentials in motoneurons and self-sustained firing in response to a transient input, such as a bout of passive stretch. These changes in reflex circuits and intrinsic properties of spinal motoneurons can lead to α-MN hyperexcitability (i.e., spontaneous motor unit firing or at subthreshold levels) and thus decreased reflex threshold.

α-MN hyperexcitability has been considered as the primary intraspinal change in stroke survivors with spasticity (Katz and Rymer, 1989). A number of studies using different technologies [surface electromyography (EMG), intramuscular EMG, and linear array surface recordings] have provided evidence of motor unit spontaneous discharges in stroke survivors with spasticity (Burne et al., 2005; Li et al., 2006; Kallenberg and Hermens, 2009, 2011; Mottram et al., 2009, 2010; Chang et al., 2013). Motor unit spontaneous discharges indicate that motor neurons are spontaneously firing. For example, spontaneous motor unit discharges were detected at rest in spastic biceps brachii muscles. The firing frequency of the spontaneous motor units was increased with increases in voluntary elbow flexion force. The spontaneous motor units continued to fire after activation despite verbal cueing to relax the muscle, although the stroke subject reported that he was relaxed (Mottram et al., 2009). The firing frequency of spontaneous units was found be greater in the post-contraction resting period than in the pre-contraction resting period (Chang et al., 2013). Collectively, these observations suggest that spontaneous motor unit discharges are likely caused by supraspinal mechanisms that are not under voluntary control (Chang et al., 2013).

Abnormal intraspinal processing and resultant α-MN hyperexcitability is likely a plastic rearrangement secondary to imbalanced excitatory and inhibitory descending inputs to the intraspinal network. Plastic rearrangement at segmental levels has been demonstrated after disruption of descending supraspinal inputs to spinal reflexes after stroke (Gracies, 2005b; Nielsen et al., 2007; Burke et al., 2013; Sist et al., 2014). Recently, Sist et al. (2014) demonstrated in an animal model that after a cortical sensorimotor stroke, there is a time-limited period of heightened post-stroke structural plasticity in both brain and spinal cord. The plastic change correlates with the severity of cortical injury and promotes behavioral recovery. Elevated structural plasticity in spinal cord is highest during the first 2 weeks and returns to baseline levels by 28 days post-stroke.

### Imbalanced Descending Regulations

Excitability of the stretch reflex pathway (afferent fibers, spinal motor neurons, and efferent fibers) is predominantly regulated by excitatory and inhibitory descending signals of supraspinal origins [see reviews in (Young, 1994; Gracies, 2005b; Sheean, 2008; Mukherjee and Chakravarty, 2010; Burke et al., 2013)]. Among five important descending pathways of the human motor system, including corticospinal (CST), RST, VST, rubrospinal, and tectospinal, the CST is the only one that originates from the cerebral cortex and is primarily involved in voluntary movement. Isolated lesions to this pathway in animal studies produce weakness, loss of dexterity, hypotonia, and hyporeflexia, instead of spasticity. In a patient with a lacunar stroke causing an exclusive lesion of the pyramidal fibers at the medullary level, no spasticity was observed (Sherman et al., 2000). The other four descending pathways, on the other hand, originate from the brainstem. The rubrospinal pathway originating from the lateral brainstem is almost absent in humans (Nathan and Smith, 1955). Tectospinal tract originates from the tectum (superior colliculus) in the midbrain and contributes to visual orientation (Brown, 1994; Sheean, 2008; Mukherjee and Chakravarty, 2010).

Reticulospinal and VST are anatomically distinct and differ in cortical control. The dorsal RST provides a powerful inhibitory effect on the spinal stretch reflex. It originates from the ventromedial reticular formation in the medulla, which receives facilitation from the motor cortex via corticoreticular fibers, and acts as the suprabulbar inhibitory system. The CST and corticoreticular tracts run adjacent to each other in the corona radiata and internal capsule. Below the medulla, the dorsal RST and the lateral CST descend adjacent to each other in the dorsolateral funiculus. In contrast, the medial RST and VST exert excitatory effects on spinal stretch reflexes. The medial RST has a diffuse origin mainly from the pontine tegmentum with efferent connections passing through and receiving contributions from the central gray and tegmentum of the midbrain and the medullar reticular formation (distinctly different from the inhibitory area). In contrast to the dorsal RST, the medial RST is not affected by stimulation of motor cortex or internal capsule. The VST originates from the lateral vestibular nucleus and descends virtually uncrossed. Both medial RST and VST descend in the ventromedial cord, anatomically distant from the lateral CST and dorsal RST in the dorsolateral cord [see reviews in Brown (1994), Young (1994), Sheean (2008), Mukherjee and Chakravarty (2010)].

**FIGURE 2:**
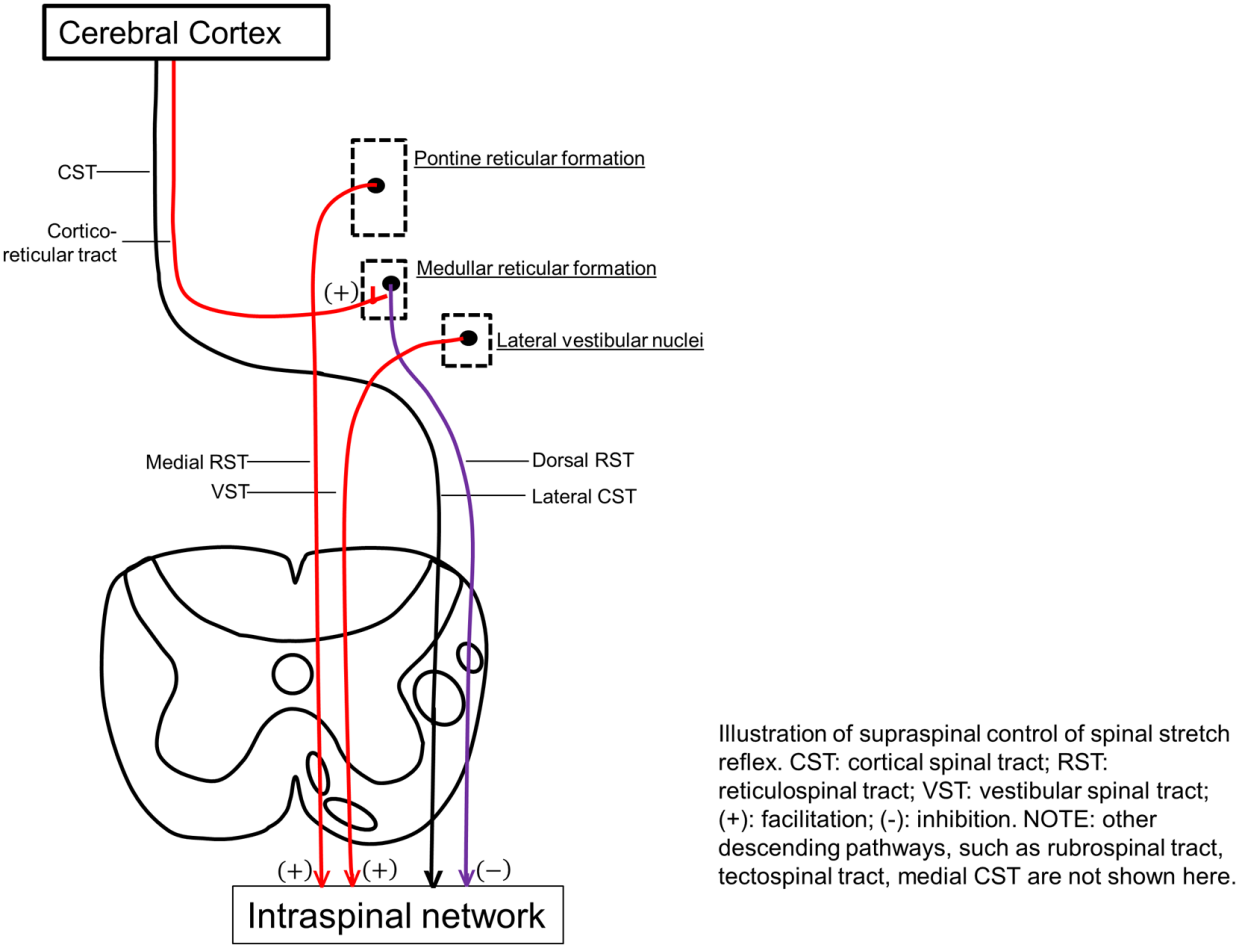
**Illustration of supraspinal control of spinal stretch reflex**. CST, cortical spinal tract; RST, reticulospinal tract; VST, vestibular spinal tract; (+), facilitation; (-), inhibition. Other descending pathways, such as rubrospinal tract, tectospinal tract, medial CST are not shown here.

Therefore, the RST and VST provide balanced excitatory and inhibitory descending regulation of the spinal stretch reflex, and any imbalance of these descending influences is thought to be a major cause of abnormal stretch reflex and thus spasticity (**Figure 2**). Abnormalities in RST outflow are considered to play a major role in the genesis of spasticity in humans, while the VST, although responsible for decerebrate rigidity, appears to have a limited role. These views are based on findings from invasive lesional studies in animals in the last century [see reviews in Brown (1994), Sheean (2008), Mukherjee and Chakravarty (2010)]. For example, section of unilateral or bilateral VST in the anterior cord only caused a transient reduction in the extensor tone in the lower limbs. With more extensive cordotomies that damage the medial RST, spasticity was drastically reduced, but tendon hyperreflexia, clonus, and adductor spasms, persisted. In stroke with cortical and internal capsular lesions, damages often happen to both CST and corticoreticular tracts, resulting in loss of cortical facilitatory input to the medullary inhibitory center. This leaves the facilitatory medial RST unopposed, since it is independent of cortical control. As a result, spastic hemiplegia with antigravity posturing is often seen.

## Recent Advances in Brainstem Mechanisms of Stretch Reflex Hyperexcitability

There is no experimental evidence from human studies, since Brown’s (1994) review of animal studies on possible brainstem mechanisms for spasticity. It is mainly due to technical difficulty to assess brainstem activity in stroke survivors with spasticity. Recent advance in functional MRI techniques with high spatial resolution offers the hope to localize brainstem nuclei (D’Ardenne et al., 2008; Katyal et al., 2010; Henderson and Macefield, 2013; Sulzer et al., 2013; Katyal and Ress, 2014). Such *in vivo* measurement of individual brainstem nuclei in stroke survivors with spasticity is probably able to provide direct evidence of pathogenesis of post-stroke spasticity. Nevertheless, recent studies have used indirect measurements to examine different brainstem mechanisms in post-stroke spasticity. Findings from these studies have provided further support for brainstem mechanisms in post-stroke spasticity.

The ASR, a brainstem-mediated reflex via reticulospinal pathways (Davis et al., 1982; Brown et al., 1991), could be used to examine reticulospinal excitability non-invasively in stroke survivors (Voordecker et al., 1997; Jankelowitz and Colebatch, 2004; Coombes et al., 2009; Honeycutt and Perreault, 2012; Li et al., 2014). Normal ASR responses could be elicited in flaccid muscles of some patients in the acute phase after cerebral infarcts, although no muscle response to magnetic cortical stimulation of the primary motor cortex was elicited (Voordecker et al., 1997). Furthermore, exaggerated ASR responses were observed in spastic muscles in chronic stroke (Jankelowitz and Colebatch, 2004). In a recent study (Li et al., 2014), we examined ASR responses in a group of chronic stroke survivors within the full spectrum of motor recovery, from stage I (flaccid) to stages II to V (spastic paresis) to stages VI and VII (full recovery) without spasticity. The rationale was that motor recovery has been arrested or plateaued in chronic stroke. ASR responses could reflect reticulospinal excitability. We observed that ASR responses were within normal limits in stroke survivors in the extreme ends of this spectrum of recovery (i.e., those who remained flaccid or have fully recovered). However, exaggerated ASR responses were consistently observed in spastic patients. These results suggest that hyperexcitability of reticulospinal pathways at rest occurs in the spastic stages, but not in the flaccid or recovered non-spastic stages. The presence of exaggerated ASR responses and hyperexcitable RST were considered to result from corticoreticular disinhibition after stroke (Voordecker et al., 1997).

Reticulospinal pathways usually have bilateral projections (Davidson and Buford, 2006; Riddle et al., 2009; Herbert et al., 2010; Baker, 2011). If RST hyperexcitability is present in stroke survivors with spasticity, unilateral voluntary activation could lead to activation on the contralateral side (i.e., motor overflow). We further compared EMG activities of resting contralateral biceps muscles between stroke survivors with and without spasticity (Li et al., 2014). During unilateral voluntary elbow flexion, EMG activity of the resting biceps muscles on the non-impaired limb increased proportionally in the spastic group, but no such correlation was found in the recovered non-spastic group. Such results of contralateral motor overflow from voluntary activation of spastic biceps muscles further support RST hyperexcitability in spastic, but not non-spastic stages. Collectively, these findings provide new evidence that RST hyperexcitability plays a critical role in pathophysiology of spasticity. Furthermore, the disappearance of spasticity and RST hyperexcitability in fully recovered stroke survivors suggests that spasticity is a phenomenon of abnormal plasticity in the course of motor recovery.

In another study that examined the potential role of VST in post-stroke spasticity, vestibular evoked myogenic potentials in the sternocleidomastoid muscle in response to high-level acoustic stimuli (130 dB) to the ears of stroke survivors were analyzed. The magnitudes of evoked potentials were greater on the impaired side than the non-impaired side, and had a strong positive relationship between the degree of asymmetry and the severity of spasticity in spastic-paretic stroke survivors, thus suggesting hyperexcitability of VST (Miller et al., 2014). Yet this level of acoustic stimuli is also likely to activate ASR via reticulospinal pathways (Davis et al., 1982; Brown et al., 1991). Furthermore, the VST mediating the evoked myogenic potentials terminates in the cervical region (Nyberg-Hansen, 1964), thus not likely to be involved in lower limb spasticity. Advanced imaging studies with *in vivo* assessment of individual brainstem nuclei will be needed to provide direct evidence of different mechanisms.

It is worth mentioning that the reticular formation is very diffusely located throughout the brainstem, yet well organized (Angeles Fernandez-Gil et al., 2010; Hurley et al., 2010). It has four longitudinal columns with ill-defined boundaries, including the paramedian, paramedian-medial, intermediate, and lateral zones. The reticular formation has connections with the spinal cord, cortices, thalamus, cerebellum, basal ganglia, and other centers in the brainstem. In addition to its above-mentioned role in the regulation of spinal reflexes, the reticular formation is also involved in the coordination of fine movements, autonomic regulation of respiration, heart rate, and blood pressure, as well as in arousal, consciousness, and modulation of pain. These anatomical connections could help understand clinical features associated with spasticity which appears to be primarily related to reticulospinal hyperexcitability (**Table 1**).

**Table 1:** Clinical features associated with post-stroke spasticity.

Associated with increased reticulospinal excitatory inputs to intraspinal network, resulting in hyperexcitable stretch reflex responses	• Increased resting tone and velocity-dependent resistance • Exaggerated response to normal stimuli (passive stretch at various speeds) or noxious stimuli (cutaneous and nociceptive) • Dynamic tone (change with posture and during walking)

Associated with imbalanced excitatory reticulospinal pathways, resulting in diffuse, stereotyped activation in the presence of diminished CST voluntary activation	• Spastic co-contraction (disordered motor control), e.g., attempt to extend the elbow leads to activation of elbow flexors → co-contraction. • Stereotyped synergy pattern (shoulder adduction, internal rotation, elbow, wrist, and finger flexion) • Associated reactions (abnormal spread of motor activities)

Associated with interactions between disinhibited reticular formation and other centers in the brainstem and cortex	• Fluctuating tone (decreased at night and during sleep) • Elevated tone with pain (via connections with reticular formation) • Elevated tone with emotional changes, such as anxiety, anger (via connections with reticular formation) • Change with respiratory activities (increased with cough; a flaccid hand opens when a patient with acute stroke yawns) • Associated with sympathetic symptoms (e.g., complex regional pain syndrome after stroke)


## Peripheral Contributions

Spasticity is one of a multitude of factors that cause hypertonia. It can be differentiated from hypertonia of other etiologies through its dependence on the speed of muscle stretch (Sheean, 2002). In a study of 24 hemiparetic patients tested within 13 months after stroke, 12 had increased resistance to passive stretch of the elbow joint among whom only five had a velocity-dependent response, i.e., spasticity (O’Dwyer et al., 1996). Furthermore, spasticity may be explained by changes in mechanical properties of muscles and not only by hyperreflexia (Dietz et al., 1981; Thilmann et al., 1991). The increased mechanical resistance may be caused by alterations in tendon compliance and physiological changes in muscle fibers. These muscular property changes may be adaptive and secondary to paresis. When a paralyzed muscle is held in a shortened position, it loses sarcomeres to “adjust” its length so that it can produce optimal force at the shortened muscle length. As a result, muscle fibers are almost twice as stiff as in normal subjects (Friden and Lieber, 2003). These changes in mechanical properties of muscles occur gradually and may lead to contracture and increased muscle stiffness (Mirbagheri et al., 2008). These components are not adequately distinguished in routine clinical examinations (Vattanasilp et al., 2000) and in quantitative assessment based on measurement of muscle stiffness in response to passive external stretch at different speeds in a laboratory setting as well (Malhotra et al., 2009).

## Implications for Clinical Assessment and Management

As discussed above, the RST hyperexcitability is likely to be a primary mechanism, while altered intraspinal network processing and peripheral muscular changes are secondary and adaptive factors that contribute to the development of post-stroke spasticity. As illustrated in **Figure 2**, the reticulospinal mechanism for post-stroke spasticity is theoretically associated with the following three pathophysiological changes along the neuroaxis: (1) disinhibited reticular formation centers in the brainstem secondary to damage of corticoreticular tract, (2) hyperexcitable descending reticulospinal projection along with diminished CST voluntary activation, (3) altered intraspinal network and hyperexcitability of spinal stretch reflex as a result. These pathophysiological changes are able to at least partly account for other clinical features associated with post-stroke spasticity, which are not as well studied as spasticity itself (**Table 1**).

### Implications for Clinical Assessments

Advances in understanding the underlying pathophysiology make it easier to understand clinical presentations and assessment of spasticity. Such clinical presentations are listed in **Table 1**. Other than its role in the regulation of muscle tone and motor function, the reticular formation has very divergent but well-organized projections to other areas/centers in the brainstem and cortex (Angeles Fernandez-Gil et al., 2010). As such, the reticular formation and its projections are involved in the regulation of other basic survival functions, such as breathing, posture, pain, temperature, and mood. RST hyperexcitability secondary to disinhibition could alter interactions among these functions. For example, recruitment of both plantar flexor and dorsiflexor muscles was observed in a stroke survivor with spasticity during normal breathing at rest, but with a predominance of plantar flexion during coughing (Gracies, 2005b). Similarly, this mechanism could also account for other common clinical observations that spasticity changes with posture (dynamic tone), temperature [weather (tighter in winter)], pain, emotion (anxiety, anger), and time (day and night fluctuation). In the experience of many clinicians, a sudden change in spasticity may result from and is a presenting sign of changes in medical conditions, among which urinary tract infections are commonly observed. These associations are usually anecdotal observations and reports. The newly proposed RST mechanism helps understand the underlying pathophysiology.

Post-stroke spasticity is traditionally examined using clinical scales such as the Ashworth scale and Tardieu scale and their variations. The RST hyperexcitability mechanism of spasticity, however, is able to offer an alternative approach to assess the severity of spasticity. Since the RST plays an important role in maintaining joint position and posture against gravity (Drew et al., 2004), its anti-gravitational force effect could lead to a shift in neuromuscular balance favoring anti-gravity muscle groups, e.g., upper limb flexors and lower limb extensors. This new balance could be reflected by a change in the resting angle of a joint, i.e., the more spastic the muscle is, the more abnormal resting angle the involved joint maintains. The concept of abnormal resting angle is particularly helpful in the clinical assessment of spasticity of small muscles or muscles that are difficult to access (e.g., sternocleidomastoid muscle). The severity of sternocleidomastoid muscle spasticity could be estimated based on abnormality of head posture.

### Implications for Spasticity Management

Disordered motor control is often seen in stroke survivors with spasticity. In patients with moderate-to-severe elbow flexor spasticity, an attempt to extend the elbow joint could lead to co-activation of the weak elbow extensors and spastic elbow flexors (Ivanhoe and Reistetter, 2004). Similarly, Kamper and Rymer (2001) reported finger flexion during an attempt to extend fingers in patients with finger flexor spasticity. The inability to activate weak extensors likely results from (1) weak extensors by diminished voluntary activation secondary to CST damage, and (2) co-activation of spastic flexors secondary to hyperexcitable RST activation after loss of inhibition. Understanding of these two separate mechanisms underlying impaired voluntary control of upper limb extensors is critical for management. Essentially, spastic flexors could be “therapeutically weakened” via botulinum toxin injection. As such, residual weak extensors may be able to function adequately, since the primary goal of the extensors is to open the hand or extend the elbow in preparation for functional operation by the flexors in most activities of daily living which does not require significant activation of the extensors (Park and Li, 2013). We name it “therapeutic weakness” with the goal of improving motor control of the antagonist.

**FIGURE 3:**
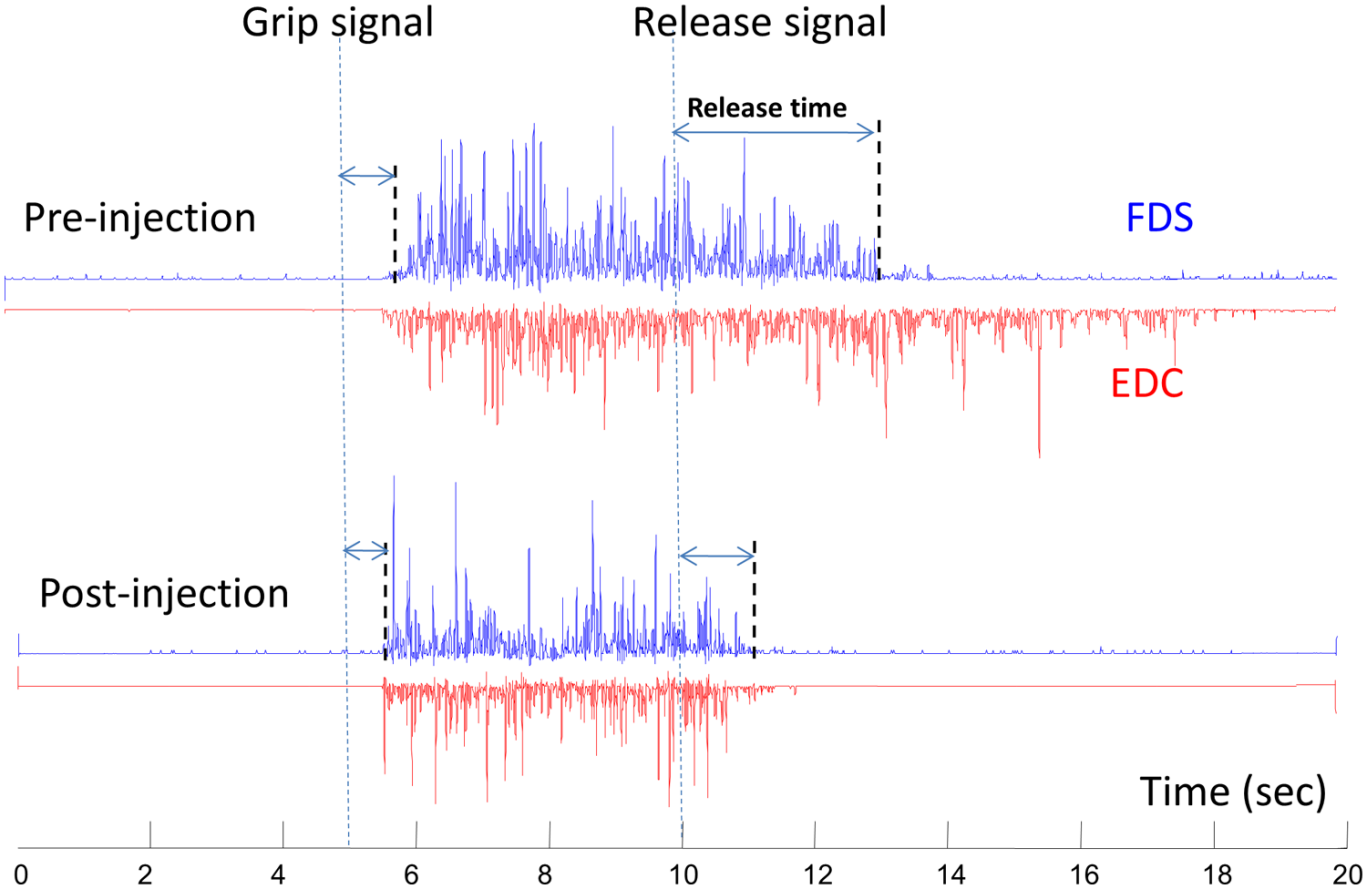
**A representative electromyography (EMG) of flexor digitorum superficialis (FDS) and extensor digitorum communis (EDC) before and 10 days after botulinum toxin injection**. The subject was asked to grip as soon and as hard as possible after the “grip signal” and relax after the “release signal” (dash lines). The release delay time decreases after injection, along with shortened EDC activities. Modified from Chang et al. (2012, with permission).

This phenomenon of therapeutic weakness is revealed in a recent case of improved voluntary grip control after botulinum toxin injection to spastic finger flexors (Chang et al., 2012). The patient was a 53-years-old female, who sustained a hemorrhagic right middle cerebral artery stroke 3 years earlier. She had finger flexor spasticity and residual weak finger/wrist extension. She received 50 units of onabotulinumtoxinA injection to each of the left flexor digitorum superficialis and flexor digitorum profundus, respectively. As expected, botulinum toxin injection led to weakness and tone reduction in the spastic finger flexors. However, she was able to open her hand faster due to improved grip release time. This was accompanied by shortened extensor electromyography activity (**Figure 3**). The improved voluntary control of hand opening/grip release was likely realized by decreased co-contraction of spastic finger flexors during voluntary finger extension. This case demonstrated that reduction in finger flexor spasticity can improve voluntary control of residual finger extension. Improvement in voluntary control of extensor muscles likely results from reduced reciprocal inhibition from the spastic flexors after injection. Previous results have shown that injections can paralyze afferent fibers (Filippi et al., 1993), in addition to blocking acetylcholine release pre-synaptically at neuromuscular junctions, as such, resulting in reduced inhibition from paralyzed flexors after injection.

This concept is further supported by another study (Bensmail et al., 2010), where 15 patients with spastic hemiparesis from stroke or traumatic brain injury were instructed to perform reaching movements within the available range of motion before and 1 month after botulinum toxin injections. Toxin was administered to the elbow, wrist, and finger flexors based on assessment of hypertonia of individual muscles. All patients were able to subsequently perform reaching movements better. Additionally, reaching velocity and smoothness improved. However, the other clinical outcomes, such as the Action Research Arm Test and the Box and Block Test remained unchanged. These findings cannot be explained by spasticity reduction alone. Though EMGs from flexors and extensors were not recorded, the authors postulated that improved reaching performance after injections to the flexors was likely related to better control of antagonist extensor muscles. In other words, voluntary control of extensor muscles during reaching movements is improved from decreased flexors spasticity and weakening of flexors after injections.

It is a commonly held clinical position that botulinum toxin injection effectively reduces spasticity, pain, and positioning (Brashear et al., 2002; Shaw et al., 2011). However, botulinum toxin injection is unlikely to improve active upper extremity function, such as reaching and grasping (Shaw et al., 2011). As discussed above, better understanding of underlying mechanisms for disordered motor control allows new therapeutic use of botulinum toxin. The finding that “therapeutic weakness” of spastic flexor muscles is associated with functional improvement is important in that it can re-shape the goals of using this therapy. In particular, “therapeutic weakness” using botulinum toxins could be considered in stroke survivors who have residual voluntary extension in the upper extremity. More research is needed to better understand this novel conceptual approach to managing spasticity.

## Concluding Remarks

Spasticity is characterized by a velocity-dependent increase in resistance during passive stretch, resulting from hyperexcitability of the stretch reflex. The underlying mechanism of the hyperexcitable stretch reflex, however, remains poorly understood. Accumulated experimental evidence has supported supraspinal origins of spasticity, likely from an imbalance between descending inhibitory and facilitatory regulation of spinal stretch reflexes secondary to cortical disinhibition after stroke. The excitability of reticulospinal and VSTs has been assessed in stroke survivors with spasticity using non-invasive indirect measures. There are strong experimental findings that support the reticulospinal hyperexcitability as a prominent underlying mechanism of post-stroke spasticity. However, the possible role of VST hyperexcitability cannot be ruled out from indirect measures. *In vivo* measure of individual brainstem nuclei in stroke survivors with spasticity using advanced fMRI techniques in the future is probably able to provide direct evidence of pathogenesis of post-stroke spasticity.

## Conflict of Interest Statement

The authors declare that the research was conducted in the absence of any commercial or financial relationships that could be construed as a potential conflict of interest.

## Abbreviations

ASR: acoustic startle reflex
α-MN: α-motor neuron
CST: corticospinal tract
RST: reticulospinal tract
UMN: upper motor neuron
VST: vestibulospinal tract
